# Meta-analysis of the association between retinopathy and the risk of stroke

**DOI:** 10.3389/fmed.2026.1887366

**Published:** 2026-06-30

**Authors:** Lingyun Wu, Weifeng Jiang

**Affiliations:** 1 The School of Medicine, Quzhou College of Technology, Quzhou, Zhenjiang, China; 2 The Department of Gerontology, the Quzhou Affiliated Hospital of Wenzhou Medical University, Quzhou People's Hospital, Quzhou, Zhenjiang, China

**Keywords:** cerebrovascular disease, meta-analysis, microvascular dysfunction, retinopathy, stroke

## Abstract

**Background:**

Retinopathy, as a manifestation of systemic microvascular dysfunction, has been increasingly recognized as a potential indicator of cerebrovascular disease. However, evidence regarding the association between retinopathy and stroke risk remains inconsistent. This meta-analysis aimed to quantitatively evaluate the relationship between retinopathy and the risk of stroke.

**Methods:**

A systematic literature search was conducted in PubMed, Embase, Cochrane Library, and CNKI from inception to June 12, 2025 to identify observational studies examining the association between retinopathy and stroke. Pooled odds ratios (ORs) with 95% confidence intervals (CIs) were calculated using a random-effects model. Due to heterogeneity in retinopathy classification, only studies with comparable exposure definitions were included in the quantitative synthesis, while others were summarized qualitatively. Study quality was assessed using the Newcastle–Ottawa Scale and the Agency for Healthcare Research and Quality checklist, as appropriate.

**Results:**

A total of 23 studies were included, of which 10 were eligible for quantitative meta-analysis. The pooled results demonstrated that retinopathy was significantly associated with an increased risk of stroke (OR = 1.87, 95% CI: 1.50–2.33). Subgroup analyses indicated a graded relationship between retinopathy severity and stroke risk, with higher risks observed in more advanced stages. The association was generally consistent across subgroups stratified by country, age, and study characteristics. Sensitivity analyses confirmed the robustness of the findings. Although no significant publication bias was detected, a potential small-study effect could not be entirely excluded.

**Conclusion:**

This meta-analysis provides evidence that retinopathy is significantly associated with an increased risk of stroke, suggesting that retinal microvascular abnormalities may represent a promising marker associated with cerebrovascular risk. However, their predictive utility for clinical risk stratification requires further validation in prospective studies. Routine retinal assessment may aid in the early identification of high-risk individuals and inform targeted prevention strategies.

## Introduction

Stroke remains a leading cause of mortality and long-term disability worldwide, imposing a substantial burden on healthcare systems and society. Globally, stroke accounts for approximately 11% of all deaths and is a major contributor to years lived with disability, particularly among older adults (1). Despite advances in prevention and treatment, early identification of individuals at high risk of stroke remains a critical challenge. Therefore, identifying accessible and reliable markers that reflect cerebrovascular risk is of considerable clinical and public health importance.

The retina and cerebral microvasculature share common embryological origins, anatomical characteristics, and physiological properties. Retinal vessels can be directly visualized non-invasively, providing a unique window into systemic microvascular health (2). Retinopathy, characterized by retinal microvascular abnormalities such as microaneurysms, hemorrhages, cotton wool spots, and arteriovenous nicking, reflects cumulative microvascular damage resulting from chronic exposure to vascular risk factors (3). Increasing evidence suggests that retinal microvascular changes may parallel pathological processes occurring in the cerebral circulation (4, 5). Several biological mechanisms have been proposed to explain the potential link between retinopathy and stroke. Retinal microvascular abnormalities are associated with endothelial dysfunction, chronic inflammation, oxidative stress, and impaired autoregulation, all of which are central to the pathogenesis of ischemic and hemorrhagic stroke (6). In addition, retinopathy frequently coexists with major vascular and metabolic risk factors, including hypertension, diabetes mellitus, dyslipidemia, and other cardiometabolic abnormalities. Although these factors may partially contribute to the observed association, retinal microvascular changes may also reflect cumulative vascular injury beyond traditional risk factor profiles (7). Moreover, retinopathy often coexists with traditional vascular risk factors, including hypertension, diabetes mellitus, and dyslipidemia, but may also capture subclinical microvascular damage beyond these established risk factors (8, 9). As such, retinopathy has been proposed as a surrogate marker of cerebral small vessel disease and overall cerebrovascular vulnerability.

Over the past two decades, numerous epidemiological studies have investigated the association between retinopathy and stroke risk in both diabetic and non-diabetic populations. While several cohort and cross-sectional studies have reported a significant association between the presence or severity of retinopathy and an increased risk of stroke, other studies have yielded null or inconsistent findings after adjustment for conventional cardiovascular risk factors (10–12). Differences in study design, population characteristics, retinopathy definitions, stroke subtypes, and confounder adjustment strategies may partly account for these discrepancies. Although some previous reviews have explored the relationship between retinal microvascular signs and cerebrovascular outcomes, a comprehensive quantitative synthesis focusing specifically on retinopathy and stroke risk remains limited. Existing meta-analyses were often constrained by a small number of studies, heterogeneous outcome definitions, or a lack of subgroup and sensitivity analyses, which may have compromised the robustness and generalizability of their conclusions (13). Furthermore, it remains unclear whether the association between retinopathy and stroke differs by diabetes status, stroke subtype, or retinopathy severity.

Therefore, we conducted a systematic review and meta-analysis to quantitatively assess the association between retinopathy and the risk of stroke. A clearer understanding of this relationship may provide insights into whether retinal microvascular abnormalities are associated with cerebrovascular risk and may inform future research evaluating their potential role in stroke risk assessment.

### Materials and methods

#### Search strategy

A comprehensive literature search was conducted to identify studies investigating the association between retinopathy and the risk of stroke. The electronic databases PubMed, Embase, Cochrane Library, and CNKI were systematically searched from inception to June 12, 2025. The search strategy combined keywords and Medical Subject Headings (MeSH) terms related to “retinopathy,” “diabetic retinopathy,” “hypertensive retinopathy,” “stroke,” “cerebrovascular disease,” and “cerebral infarction.” No restrictions on language or geographic region were applied. In addition, the reference lists of included articles and relevant reviews were manually screened to identify any additional eligible studies. The detailed search strategy for each database is provided in Supplementary Table S1.

### Inclusion and exclusion criteria

Studies were included if they met the following criteria: Study Design: Cohort, case-control, or cross-sectional studies. Population: Adult participants. Exposure: Presence of retinopathy, as defined in the original studies using standardized diagnostic methods such as fundus photography, ophthalmologic examination, retinal imaging, or medical record documentation. Where available, established grading systems such as the Keith–Wagener–Barker (KWB) classification or the Early Treatment Diabetic Retinopathy Study (ETDRS) scale were applied. Studies reporting retinopathy as a binary variable (present vs absent) were also included. Comparison: Participants without retinopathy. Outcome: Incidence or prevalence of stroke. Studies were excluded if they met any of the following criteria: (1). Were reviews, case reports, conference abstracts, editorials, or animal studies; (2). Reported outcomes not directly relevant to incident stroke, such as stroke recurrence, composite outcomes, or stroke-related mortality; (3). Lacked sufficient data to extract or calculate effect estimates. (4). Lacked an appropriate comparison group. (5). Included duplicate or overlapping populations, in which case the most comprehensive or most recent study was retained.

### Data extraction and quality assessment

Two reviewers (Lingyun Wu and Weifeng Jiang) independently screened titles and abstracts, followed by full-text assessments to determine study eligibility. Data extraction was conducted using a standardized form, capturing the following information: first author, year of publication, country, study design, sample size, participants, age, diagnostic, methods of retinopathy, grading criteria for retinopathy, stroke type, first stroke, diagnostic methods of stroke, covariates, and effect estimates (ORs, RRs, or HRs) with corresponding 95% CIs. The methodological quality of the included studies was independently assessed by two reviewers using appropriate tools according to study design. For cohort and case–control studies, the Newcastle–Ottawa Scale was applied (14), which evaluates three domains: selection, comparability, and outcome/exposure. Studies scoring ≥6 were considered to be of high quality. For cross-sectional studies, the Agency for Healthcare Research and Quality methodology checklist was used to assess methodological quality (15). This checklist comprises 11 items evaluating key aspects such as study design, data collection, and control of bias. Each item was scored as “1” (yes) or “0” (no/unclear), with a total score ranging from 0 to 11. Higher scores indicated better methodological quality. Any disagreements between reviewers were resolved through discussion or consultation with a third reviewer.

### Statistical analysis

The association between retinopathy and the risk of stroke was quantified by pooling effect estimates with corresponding 95% confidence intervals (CIs). Given the anticipated clinical and methodological heterogeneity across studies, a random-effects model was applied. Due to substantial heterogeneity in retinopathy classification and exposure definitions, quantitative synthesis was restricted to studies that compared the presence of retinopathy vs. its absence using a binary exposure definition and reported a corresponding overall effect estimate for stroke risk. Studies were excluded from the meta-analysis when they exclusively reported associations according to multiple retinopathy severity categories without providing a comparable overall effect estimate, used unique grading systems that could not be harmonized across studies, or adopted reference groups that were not comparable to other studies (e.g., comparisons between different retinopathy grades rather than retinopathy vs. no retinopathy). These criteria were specified during the review process before data synthesis to improve methodological comparability and reduce exposure misclassification.

Effect estimates reported as odds ratios (ORs), risk ratios (RRs), and hazard ratios (HRs) were extracted from the most fully adjusted models whenever available. Stroke event frequencies were extracted when reported and are summarized in Supplementary Table S3. Although outcome frequencies varied across studies, stroke event rates were generally low in most studies. Therefore, ORs, RRs, and HRs were treated as comparable relative effect measures, transformed to the natural logarithmic scale, and pooled using the generic inverse-variance method under a random-effects model without further conversion. To assess the potential influence of effect measure type on the pooled results, an additional subgroup analysis stratified by HR/RR vs. OR was conducted.

Statistical heterogeneity was assessed using Cochran's Q test (with *p* < 0.10 indicating significant heterogeneity) and quantified using the I^2^ statistic, where values >50% were considered indicative of substantial heterogeneity. Sensitivity analyses were performed by sequentially omitting individual studies to evaluate the robustness and stability of the pooled estimates. Subgroup analyses were conducted, where data permitted, according to grading criteria for retinopathy, country, participants, age, and first stroke, to explore potential sources of heterogeneity. Potential publication bias was assessed through visual inspection of funnel plots, complemented by Egger's regression asymmetry test. All statistical analyses were conducted using STATA software (version 18.0). A two-sided *p*-value < 0.05 was considered statistically significant, unless otherwise specified. This systematic review and meta-analysis was not prospectively registered in PROSPERO or any other review registry.

## Result

### Study selection and study characteristics

**Figure 1:**
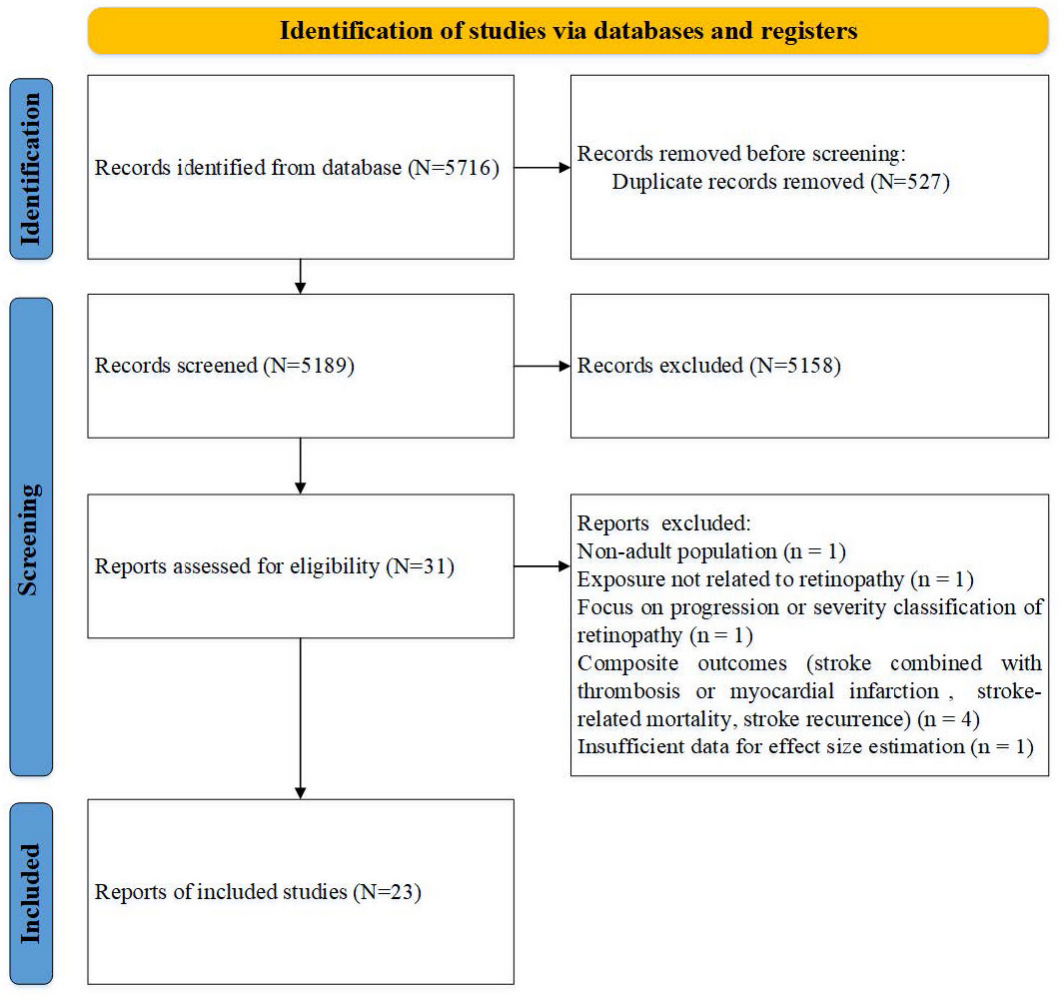
A flow diagram demonstrating the study selection process.

A total of 5,716 records were identified through database searching. After removing duplicates (*n* = 527), 5,189 articles remained for title and abstract screening. Based on inclusion and exclusion criteria, 31 full-text articles were assessed for eligibility, and finally, 23 studies were included in the meta-analysis (Figure 1). The included studies were published between 1995 and 2024, comprising a total of 138,296 adult participants. Study designs included 6 Cross-sectional studies, 13 prospective cohort studies, 3 retrospective cohort studies, and 1 case-control studies. Retinopathy was assessed using standardized methods such as fundus photography, retinal imaging, ophthalmologic examination, or medical record documentation. Several studies applied established grading systems, including the Keith–Wagener–Barker (KWB) classification and the Early Treatment Diabetic Retinopathy Study (ETDRS) scale, while others reported retinopathy as a binary exposure without grading. Stroke outcomes included ischemic stroke, hemorrhagic stroke, or composite definitions of any stroke, and were ascertained using hospital records, national registries, imaging modalities (CT/MRI), or self-reported physician diagnoses. Most studies focused on incident stroke, although a few did not clearly specify whether first-ever stroke events were exclusively considered. With respect to confounding control, the majority of studies adjusted for key demographic and cardiovascular risk factors, including age, sex, body mass index, blood pressure, smoking status, lipid profiles, and diabetes-related variables. The methodological quality of the included studies was generally moderate to high. According to the Newcastle–Ottawa Scale, cohort studies achieved scores ranging from 6 to 9, with most studies rated as high quality (NOS ≥7). Cross-sectional studies, assessed using the Agency for Healthcare Research and Quality methodology checklist, had scores ranging from 6 to 7 out of a maximum of 11, indicating moderate methodological quality (Tables 1, 2). All studies included in the quantitative meta-analysis met the predefined quality criteria and were therefore considered to be of moderate-to-high methodological quality. Of the 23 included studies, 10 provided sufficiently comparable classifications of retinopathy and were therefore included in the quantitative meta-analysis, while the remaining studies were not pooled due to substantial heterogeneity in retinopathy definitions and grading systems.

**Table 1:** Characteristics of eligible studies.

References	Country	Design	Sample size	Quality score
Suri (16)	USA	Prospective cohort	4,753	7
Ong (17)	Singapore	Prospective cohort	2,907	8
Harbaoui (18)	France	Prospective cohort	1,031	7
Thiagarajah (19)	Malaysia	Cross-sectional	101	6
Chen (20)	China	Prospective cohort	9,793	8
Zhao (21)	China	Cross-sectional	86	6
Li (22)	China	Cross-sectional	100	6
Li (23)	Japan	Prospective cohort	7,027	8
Petitti (24)	USA	Case-control	104	6
Klein (25)	USA	Prospective cohort	918	7
Cheung (26)	Australia	Prospective cohort	1,546	8
Hägg (27)	Finland	Prospective cohort	4,083	7
Wong (28)	USA	Prospective cohort	2,828	8
Yang (29)	USA	Prospective cohort	21,049	6
Drinkwater (30)	Australia	Prospective cohort	1,473	8
Hsu (31)	China	Retrospective cohort	13,220	7
Modjtahedi (32)	USA	Retrospective cohort	77,376	8
Eriksson (33)	Finland	Retrospective cohort	1,268	8
Tang (34)	China	Cross-sectional	1,948	7
Grunwald (35)	USA	Cross-sectional	1,936	7
Grunwald (36)	USA	Prospective cohort	1,245	8
Wong (37)	USA	Prospective cohort	1,684	9
Hughes (3)	UK	Cross-sectional	1,185	8

**Table 2:** Characteristics of participants.

Author	Participants	Age	Diagnostic methods of retinopathy	Grading criteria for retinopathy	Stroke type	First stroke	Diagnostic methods of stroke	Covariates
Suri	Hypertension	49.8	Standardized ophthalmologic examination	Not graded	Ischemic stroke	Yes	Hospital records and death certificates with physician adjudication	Age, sex, race/ethnicity, body mass index (BMI), smoking, systolic blood pressure (SBP), serum cholesterol, diabetes
Ong	Hypertension	60.5	Retinal photography	Wong and Mitchell classification	Ischemic and hemorrhagic stroke	Yes	Active surveillance with hospital record review and physician adjudication (ICD-9-CM)	Age, sex, race-center, mean arterial pressure, fasting glucose, HDL cholesterol, triglycerides, BMI, smoking, alcohol use
Harbaoui	Hypertension	44.0	Ophthalmoscopic fundus examination	KWB classification	Fatal stroke	No	Mortality registry and death certificates (ICD-9)	Age, sex, cardiovascular disease history, estimated glomerular filtration rate (eGFR), BMI, diabetes, smoking, antihypertensive treatment, mean blood pressure
Thiagarajah	Hypertension	54.0	Fundus photography	Wong and Mitchell classification	Hemorrhagic stroke	Not reported	Cranial CT	Not reported
Chen	Hypertension	63.3	Fundus photography	KWB classification	Ischemic, hemorrhagic, and unspecified stroke	Yes	CDC registries and hospital records (ICD-10)	Age, sex, BMI, SBP, DBP, diabetes, triglycerides, total cholesterol, HDL, folic acid, homocysteine, fasting glucose, creatinine, treatment group, MTHFR genotype, smoking, alcohol
Zhao	Hypertension	62.9	Fundus photography	KWB classification	Ischemic and hemorrhagic stroke	Not reported	Cranial CT and MRI	Age, hypertension duration, hypertension grade, arteriolar-to-venular ratio
Li	Hypertension	63.9	Fundus photography	KWB classification	Ischemic and hemorrhagic stroke	Not reported	Cranial CT and MRI	Same as above
Li	Hypertension	54.3	Retinal photography	KWB classification	Ischemic and hemorrhagic stroke	Yes	Medical records with CT/MRI or autopsy confirmation	BMI, SBP, antihypertensive use, cholesterol, HDL, lipid-lowering therapy, atrial fibrillation, LVH, eGFR, diabetes, smoking, alcohol
Petitti	Diabetes	67.0	Medical records	Not graded	Ischemic stroke	Yes	Hospital discharge records with chart review	Age, sex, smoking, insulin use, SBP, glucose
Klein	Diabetes	Not reported	Fundus photography	ETDRS scale	Any stroke	Yes	Self-reported physician diagnosis	Age, sex, HbA1c, hypertension, neuropathy, aspirin use
Cheung	Diabetes	60.12	Fundus photography	ETDRS scale	Ischemic stroke	Yes	Annual follow-up and hospital record review	Age, sex, race, blood pressure, glucose, insulin, diabetes duration, lipids, smoking
Hägg	Diabetes	37.41	History of retinal laser treatment	Not graded	Ischemic and hemorrhagic stroke	Not reported	National registers and questionnaires with medical verification	Age, sex, SBP, DBP, BMI, lipids, smoking
Wong	Diabetes	62.1	Fundus photography	ETDRS scale	Ischemic and hemorrhagic stroke	No	Physician adjudication	Age, race, sex, smoking, HbA1c, diabetes duration, cholesterol, CVD history
Yang	Diabetes	Not reported	Self-reported	Not graded	Any stroke	No	Self-reported	Age, sex, education, smoking, BMI, hypertension, hyperlipidemia
Drinkwater	Diabetes	65.4	Fundus photography	ETDRS scale	Ischemic and hemorrhagic stroke	Yes	Hospital and death registry records	Age, HbA1c, smoking, NT-proBNP, vascular disease
Hsu	Diabetes	Not reported	Insurance claims (ICD-9-CM)	ETDRS scale	Ischemic stroke	Yes	Insurance claims (ICD-9-CM)	Age, sex, comorbidities, medications
Modjtahedi	Diabetes	59.8	Fundus photography	ETDRS scale	Any stroke	Yes	Administrative claims (ICD-9/10)	Age, sex, race, HbA1c, diabetes duration, lipids, BP, smoking
Eriksson	Diabetes	38.7	Ophthalmologic records	ETDRS scale	Ischemic and hemorrhagic stroke	Yes	Hospital registry with verification	Age, sex, diabetes duration, LDL, SBP, kidney disease
Tang	Diabetes	62.8	Digital retinal imaging	ETDRS scale	Any stroke	Not reported	Self-reported physician diagnosis	Age, sex, education, BMI, smoking, hypertension
Grunwald	Renal insufficiency	58.3	Fundus photography	ETDRS scale	Any stroke	No	Questionnaires with clinical confirmation	Age, sex, lipids, SBP, smoking, diabetes, eGFR
Grunwald	Renal insufficiency	57.7	Fundus photography	ETDRS scale	Any stroke	Yes	Same as above	Same as above
Wong	General adults	62.2	Retinal photography	Not graded	Ischemic and hemorrhagic stroke	Yes	Interviews, hospital records, death certificates	Age, sex, BP, diabetes, cholesterol, smoking
Hughes	General adults	69.0	Fundus photography	NHSDESP classification	Cerebral infarction and stroke	No	Brain MRI	Age, sex, ethnicity, hypertension, BMI, HbA1c, cholesterol, CRP, smoking

### Qualitative synthesis of studies not included in the meta-analysis

Thirteen studies were not included in the quantitative synthesis because their retinopathy exposure definitions, grading systems, and comparison categories were not sufficiently comparable for meta-analysis. A structured summary of these studies is provided in Supplementary Table S2. Among studies of hypertensive retinopathy, most reported a positive association between increasing retinopathy severity and stroke risk. For example, Ong et al. (17), Harbaoui et al. (18), and Chen et al. (20). Observed progressively higher stroke risks among participants with more advanced hypertensive retinopathy. Zhao et al. (21), and Li et al. (23) also reported increased stroke risk in higher-grade retinopathy categories compared with lower-grade categories. Although Thiagarajah et al. (19). Did not perform multivariable regression analyses, the crude stroke prevalence increased substantially with greater retinopathy severity. Similarly, studies using ETDRS-based diabetic retinopathy classifications generally demonstrated a graded relationship between retinopathy severity and stroke risk. Modjtahedi et al. (32)., Eriksson et al. (33)., Tang et al. (34)., Grunwald et al. (36)., and Hughes et al. (3). Consistently reported higher stroke risks among participants with proliferative or advanced retinopathy compared with those without retinopathy or with milder disease. Although effect estimates varied across studies and some confidence intervals included the null value, none of the studies suggested a protective association between retinopathy and stroke. Overall, the qualitative evidence from studies not included in the meta-analysis was directionally consistent with the pooled findings, supporting a positive association between retinopathy severity and stroke risk.

### Synthesis of results

**Figure 2:**
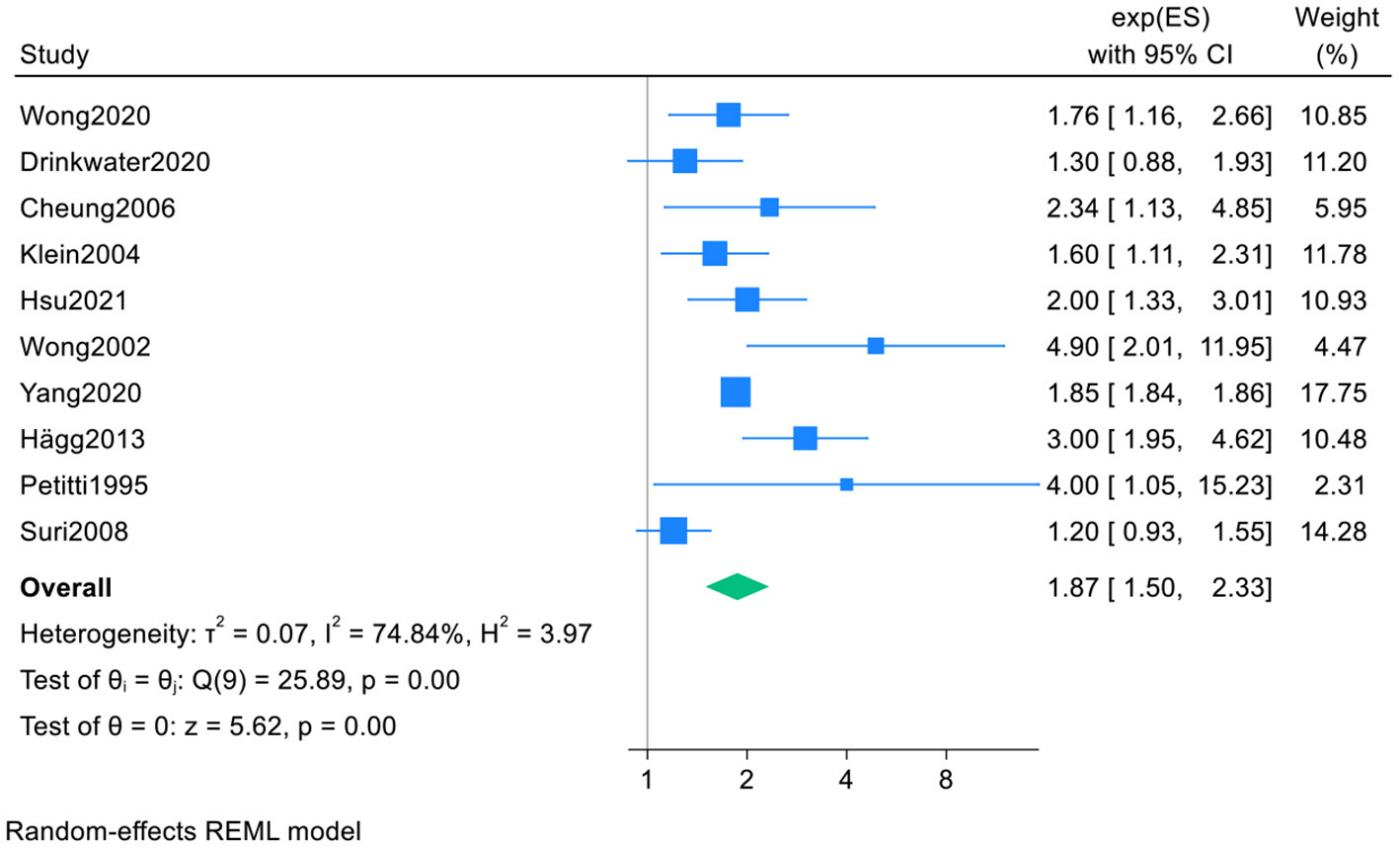
Forest plot of the effect of retinopathy on the risk of stroke.

The pooled analysis demonstrated that retinopathy was significantly associated with an increased risk of stroke. The overall effect estimate was OR = 1.87 (95% CI: 1.50–2.33, *p* < 0.001) (Figure 2), indicating that individuals with retinopathy had an approximately 87% higher risk of developing stroke compared with those without retinopathy. However, substantial heterogeneity was observed across studies (I^2^ = 74.84%, *p* < 0.001), suggesting variability in study populations, exposure assessment, and outcome definitions. To explore potential sources of heterogeneity, subgroup analyses were conducted based on retinopathy grading criteria, country, participant characteristics, age, and whether first-ever stroke events were assessed (Table 3). Notably, the magnitude of the association tended to increase across retinopathy severity categories. Studies using the Keith–Wagener–Barker (KWB) classification showed that mild retinopathy (Grade 1–2) was associated with a modest increase in stroke risk (OR = 1.30), whereas severe retinopathy (Grade 3–4) was associated with a substantially higher risk (OR = 2.65). Similarly, studies applying the Early Treatment Diabetic Retinopathy Study (ETDRS) scale demonstrated progressively increasing risks from non-proliferative diabetic retinopathy (NPDR) (OR = 1.50) to proliferative diabetic retinopathy (PDR) (OR = 3.00). Subgroup analyses further indicated that the association was consistent across different countries and age groups, with no statistically significant differences observed between subgroups (all p for interaction > 0.05). Among studies involving patients with diabetes, the association remained statistically significant (OR = 1.87, 95% CI: 1.61–2.17), with relatively low between-study heterogeneity (I^2^ = 32.4%). Stroke event frequencies were available for eight of the ten studies included in the quantitative synthesis and ranged from 1.9 to 9.8% (Supplementary Table S4). Event rates among studies reporting HRs or RRs ranged from 1.9 to 6.5%, while those among OR-based studies were 6.0% and 9.8%, respectively. Given the generally low incidence of stroke in most studies and the comparable results observed in the effect-measure-specific subgroup analyses, the overall findings were considered unlikely to be materially influenced by differences in effect measure type. Subgroup analyses also indicated that the association between retinopathy and stroke was consistent across different effect measure types (HR, RR, and OR), suggesting that the pooled results were robust and not materially influenced by the choice of effect size metric (Supplementary Figure S1).

**Table 3:** Subgroup analysis.

Subgroup	Group	No. studies	Overall effect	Heterogeneity			
			OR (95% CI)	Z-score	*p*-value	*I^2^* (%)	*p*-value
Grading criteria for retinopathy	KWB classification: No DR vs. Grade 1–2	3	1.30 (1.11, 1.52)	3.24	< 0.001	0.00	0.47
	KWB classification: No DR vs. Grade 3–4	2	2.65 (1.75, 3.93)	4.65	< 0.001	0.00	0.48
	ETDRS Scale: No DR vs. NPDR	5	1.50 (1.38, 1.63)	9.53	< 0.001	0.00	0.52
	ETDRS Scale: No DR vs. PDR	4	3.00 (2.38, 3.79)	9.27	< 0.001	29.74	0.22
Country	United States	6	1.76 (1.37, 2.26)	4.37	< 0.001	71.30	0.00
	Australia	2	1.61 (0.92, 2.79)	1.68	0.093	48.10	0.17
	China	1	2.00 (1.33, 3.01)	3.33	0.001	–	–
	Finland	1	3.00 (1.95, 4.61)	4.99	< 0.001	–	–
Population characteristics	Diabetes	8	1.87 (1.61, 2.17)	8.29	< 0.001	32.40	0.17
	Hypertension	1	1.20 (0.93, 1.55)	1.40	0.162	–	–
	General adults	1	4.89 (2.01, 11.96)	3.49	< 0.001	–	–
Age	≥60 years	5	2.12 (1.37, 3.27)	3.37	0.001	57.10	0.05
	< 60 years	2	1.87 (0.76, 4.58)	1.36	0.173	92.20	0.00
First stroke	Yes	8	1.96 (1.44, 2.68)	4.23	< 0.001	70.70	0.00
	No	2	1.84 (1.83, 1.86)	221.82	< 0.001	0.00	0.82

### Sensitivity analysis

**Figure 3:**
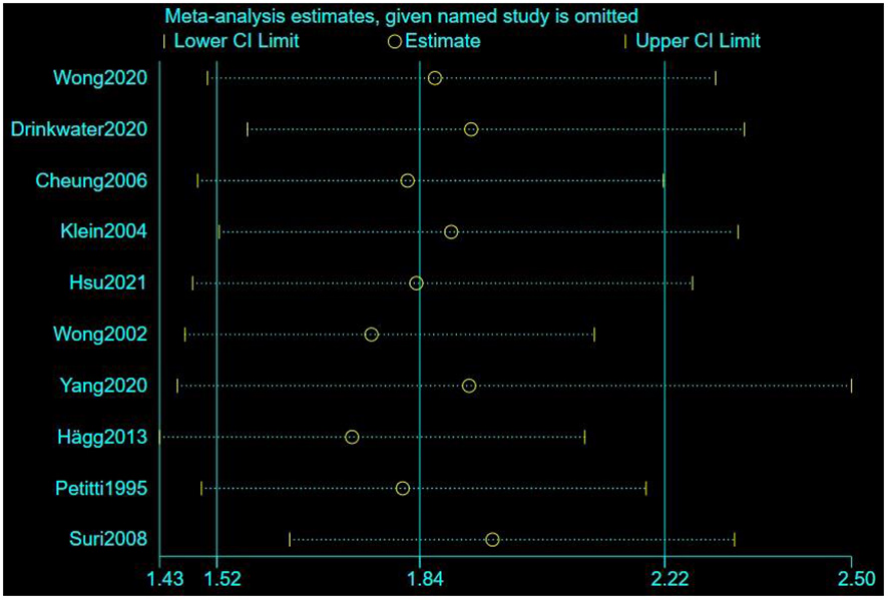
Sensitivity analysis of the stroke risk.

Sensitivity analyses were performed by sequentially excluding each individual study to assess the robustness of the pooled results. The recalculated effect estimates remained largely unchanged after the removal of any single study, indicating that no individual study disproportionately influenced the overall findings (Figure 3). These results suggest that the observed association between retinopathy and stroke risk is stable and robust, despite the presence of between-study heterogeneity.

### Publication bias

**Figure 4:**
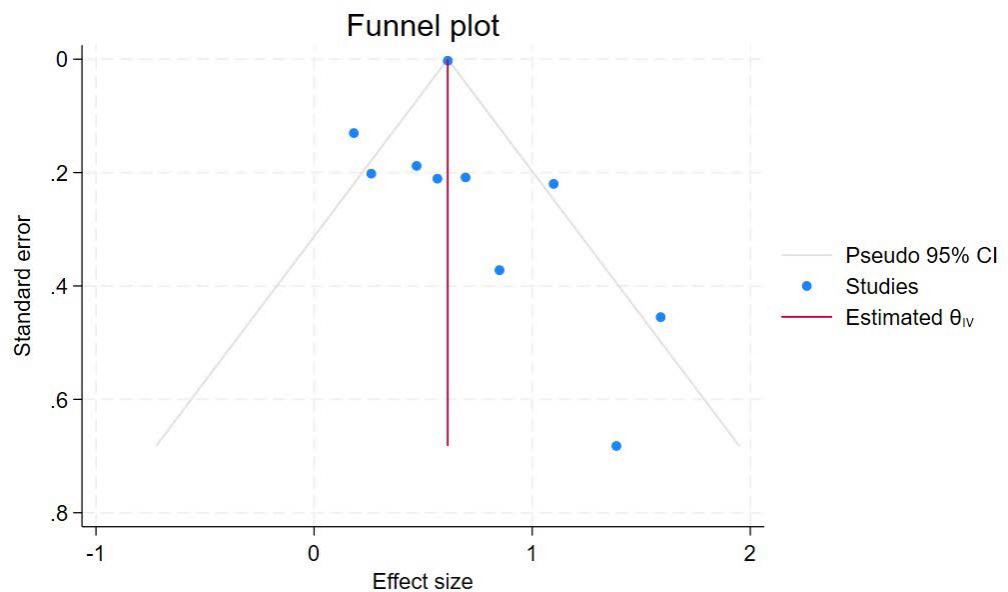
Funnel plot of the stroke risk.

Publication bias was assessed using funnel plot visualization and Egger's regression test. Visual inspection of the funnel plot revealed a generally symmetrical distribution of studies (Figure 4), although a slight asymmetry could not be entirely excluded. Consistent with this observation, Egger's test did not indicate statistically significant publication bias (*p* = 0.0606), although the result was close to the conventional threshold for significance. Taken together, these findings suggest that there is no strong evidence of publication bias, but the possibility of a small-study effect cannot be completely ruled out.

## Discussion

In this meta-analysis, we found that retinopathy was significantly associated with an increased risk of stroke, with individuals exhibiting retinopathy having an approximately 87% higher risk compared with those without retinopathy. Subgroup analyses demonstrated generally consistent results across different populations, countries, and study characteristics, with no statistically significant differences observed between most subgroups. Importantly, sensitivity analyses confirmed the robustness of our findings, as the pooled estimates remained stable after sequential exclusion of individual studies.

A particularly noteworthy finding of this study is that more severe categories of retinopathy were generally associated with higher stroke risk estimates. Across both the KWB and ETDRS classification systems, studies consistently reported larger effect estimates for advanced retinopathy than for milder forms, suggesting a severity-dependent association. However, because a formal dose–response meta-analysis was not performed, these findings should be interpreted as observational evidence across severity categories rather than definitive evidence of a dose–response relationship. Both the Keith–Wagener–Barker (KWB) classification and the Early Treatment Diabetic Retinopathy Study (ETDRS) scale consistently showed that more advanced stages of retinopathy were associated with progressively higher risks of stroke. This gradient pattern strengthens the evidence for a potential causal relationship and suggests that retinal microvascular damage may reflect the systemic vascular burden contributing to cerebrovascular events (38).

Our findings are consistent with several large-scale cohort studies reporting a positive association between retinal microvascular abnormalities and incident stroke (10, 11). However, several studies reported weaker or non-significant associations. For example, Ong et al. (17). Found that mild hypertensive retinopathy was not significantly associated with stroke risk after adjustment for multiple cardiovascular risk factors, whereas moderate-to-severe retinopathy remained significant. Similarly, studies from the Chronic Renal Insufficiency Cohort reported non-significant associations for mild or moderate non-proliferative retinopathy after adjustment for kidney function and other vascular risk factors, while proliferative retinopathy remained strongly associated with stroke risk (35, 36). In addition, Harbaoui et al. (18). Reported a weaker association for mild retinopathy, whereas severe retinopathy remained significantly associated with stroke risk. These findings suggest that differences in population characteristics, outcome definitions, disease severity, and the extent of confounder adjustment may contribute to the variability observed across studies. Compared with previous studies focusing primarily on traditional cardiovascular risk factors, our meta-analysis highlights the importance of retinal microvascular changes as an accessible and non-invasive biomarker for stroke risk stratification (39). By incorporating studies from diverse populations and clinical settings, our analysis provides broader evidence supporting this association, although such diversity may also contribute to the heterogeneity observed across studies.

Several biological mechanisms may underlie the observed relationship. Retinopathy is widely regarded as a manifestation of systemic microvascular dysfunction (40). Structural and functional alterations in retinal vessels—such as arteriolar narrowing, microaneurysms, and endothelial dysfunction—may mirror similar pathological processes occurring in the cerebral microcirculation (4). Endothelial dysfunction, chronic inflammation, oxidative stress, and impaired autoregulation of blood flow have all been implicated in both retinal and cerebral vascular damage (41). In addition, shared risk factors, including hypertension, diabetes, and dyslipidemia, may further contribute to the progression of both retinopathy and cerebrovascular disease (38). Therefore, retinopathy may serve not only as a local ocular condition but also as a surrogate marker of systemic vascular pathology (42). From a clinical perspective, the observed association highlights the potential value of retinal microvascular assessment as an indicator of systemic vascular health (2). Because retinal imaging is non-invasive and widely available, retinal abnormalities may help identify individuals who warrant closer cardiovascular evaluation (43). However, the present findings do not establish the predictive performance of retinopathy for stroke risk, nor do they demonstrate that incorporating retinal assessment into existing risk prediction models improves clinical decision-making (44). Further prospective studies evaluating discrimination, calibration, and risk reclassification metrics are needed before retinal assessment can be recommended as a routine tool for stroke risk stratification.

Several limitations of this study should be acknowledged. First, most included studies were observational in nature, and although many adjusted for major confounders, residual confounding cannot be entirely excluded. Second, substantial heterogeneity was observed across studies, which may be attributed to differences in study populations, retinopathy assessment methods, and outcome definitions. Although the inclusion of studies from diverse populations and clinical settings enhances the generalizability of our findings, it may also contribute to increased between-study heterogeneity and reduce the consistency of pooled estimates. Third, only a subset of the included studies was eligible for quantitative synthesis due to heterogeneity in retinopathy classification, which may limit the generalizability of the pooled estimates but was necessary to ensure methodological comparability and reduce misclassification bias. Fourth, variability in diagnostic criteria and grading systems for retinopathy may have introduced measurement bias. Fifth, although Egger's test did not indicate statistically significant publication bias, the borderline result suggests that potential small-study effects cannot be completely ruled out. Fourth, some studies did not clearly distinguish between first-ever and recurrent stroke, which may have influenced the pooled estimates. Sixth, this review was not prospectively registered in a publicly accessible database such as PROSPERO. Therefore, although subgroup analyses were planned during the review process, the distinction between prespecified and *post hoc* analyses cannot be fully verified, which may increase the risk of selective reporting. In addition„ although most studies reported relatively low stroke event rates, outcome frequencies were not consistently available across all included studies. One study did not report stroke event numbers, and another adopted a case-control design in which incidence rates could not be estimated. Therefore, the assumption that ORs, RRs, and HRs are approximately comparable may not apply equally across all studies. Nevertheless, subgroup analyses according to effect measure type yielded similar pooled estimates, supporting the robustness of the overall findings. Furthermore, we did not perform a formal GRADE assessment of the certainty of evidence, which may limit the evaluation of the overall strength of the findings. Future research should focus on large-scale prospective cohort studies with standardized assessment of retinopathy and stroke outcomes, as well as mechanistic investigations to further elucidate the causal pathways linking retinal microvascular abnormalities to cerebrovascular disease.

## Conclusion

This meta-analysis demonstrates that retinopathy is significantly associated with an increased risk of stroke, with evidence suggesting that more severe retinopathy is associated with higher stroke risk. These findings support the role of retinal microvascular abnormalities as potential indicators of systemic vascular dysfunction and cerebrovascular risk. From a clinical perspective, retinal assessment may provide complementary information regarding systemic vascular damage and cerebrovascular risk. Whether routine retinal examination improves stroke risk prediction beyond established cardiovascular risk factors remains uncertain and warrants further investigation. Further well-designed prospective studies with standardized diagnostic criteria are warranted to confirm these findings and to clarify the underlying mechanisms linking retinopathy to stroke.

## Data Availability

The original contributions presented in the study are included in the article/Supplementary material, further inquiries can be directed to the corresponding author.

## References

[1] Feigin VL Brainin M Norrving B Martins SO Pandian J Lindsay P . World stroke organization: global stroke fact sheet 2025. Int J Stroke. (2025) 20:132–44. doi: 10.1177/17474930241308142 39635884 PMC11786524

[2] Girach Z Sarian A Maldonado-García C Ravikumar N Sergouniotis PI Rothwell PM . Retinal imaging for the assessment of stroke risk: a systematic review. J Neurol. (2024) 271:2285–97. doi: 10.1007/s00415-023-12171-6 38430271 PMC11055692

[3] Hughes AD Falaschetti E Witt N Wijetunge S Thom SA Tillin T . Association of retinopathy and retinal microvascular abnormalities with stroke and cerebrovascular disease. Stroke. (2016) 47:2862–4. doi: 10.1161/STROKEAHA.116.014998 27729577 PMC5082730

[4] Moss HE. Retinal vascular changes are a marker for cerebral vascular diseases. Curr Neurol Neurosci Rep. (2015) 15:40. doi: 10.1007/s11910-015-0561-1 26008809 PMC4743651

[5] Wu N Xu M Chen S Wu S Li J Hui Y . Retinal vascular morphology reflects and predicts cerebral small vessel disease: evidences from eye-brain imaging analysis. Research. (2025) 8:0633. doi: 10.34133/research.0633 40052159 PMC11883085

[6] Liu X Chang Y Li Y Liu Y Song W Lu J . Oxidative stress and retinopathy: evidence from epidemiological studies. J Transl Med. (2025) 23:94. doi: 10.1186/s12967-025-06110-4 39838377 PMC11748554

[7] Li Z Yuan Y Qi Q Wang Q Feng L. Relationship between dyslipidemia and diabetic retinopathy in patients with type 2 diabetes mellitus: a systematic review and meta-analysis. Syst Rev. (2023) 12:148. doi: 10.1186/s13643-023-02321-2 37620980 PMC10463379

[8] Rao H Jalali JA Johnston TP Koulen P. Emerging roles of dyslipidemia and hyperglycemia in diabetic retinopathy: molecular mechanisms and clinical perspectives. Front Endocrinol. (2021) 12:620045. doi: 10.3389/fendo.2021.620045 33828528 PMC8020813

[9] Chen X Zhang X Gong Z Yang Y Zhang X Wang Q . The link between diabetic retinal and renal microvasculopathy is associated with dyslipidemia and upregulated circulating level of cytokines. Front Public Health. (2022) 10:1040319. doi: 10.3389/fpubh.2022.1040319 36733289 PMC9886881

[10] Wong TY Klein R Couper DJ Cooper LS Shahar E Hubbard LD . Retinal microvascular abnormalities and incident stroke: the atherosclerosis risk in communities study. Lancet. (2001) 358:1134–40. doi: 10.1016/S0140-6736(01)06253-5 11597667

[11] Kawasaki R Xie J Cheung N Lamoureux E Klein R Klein BE . Retinal microvascular signs and risk of stroke: the multi-ethnic study of atherosclerosis (MESA). Stroke. (2012) 43:3245–51. doi: 10.1161/STROKEAHA.112.673335 23111439 PMC3508325

[12] Wang W Zhang TH Jia L. Cross-sectional study on the association between retinal microcirculation changes based on optical coherence tomography angiography and mild cognitive impairment in patients with type 2 diabetes. Front Med. (2025) 12:1579562. doi: 10.3389/fmed.2025.1579562 40761848 PMC12318948

[13] Wang Z Feng L Wu M Ding F Liu C Xie G . Hypertensive retinopathy can predict stroke: A systematic review and meta-analysis based on observational studies. J Stroke Cerebrovasc Dis. (2024) 33:107953. doi: 10.1016/j.jstrokecerebrovasdis.2024.107953 39227002

[14] Wells GA Shea B O'Connell D Peterson J Welch V Losos M . The Newcastle-Ottawa Scale (NOS) for Assessing the Quality of Non-Randomised Studies in Meta-Analyses Ottawa: Ottawa Hospital Research Institute. (2014). Available online at: http://www.ohri.ca/programs/clinical_epidemiology/oxford.asp

[15] Quality AfHRa. Methods Guide for Effectiveness and Comparative Effectiveness Reviews.Rockville. Rockville, MD: AHRQ (2012).

[16] Suri MF Qureshi AI. Hypertensive retinopathy and risk of cardiovascular diseases in a national cohort. J Vasc Interv Neurol. (2008) 1:75–8. 22518227 PMC3317297

[17] Ong YT Wong TY Klein R Klein BE Mitchell P Sharrett AR . Hypertensive retinopathy and risk of stroke. Hypertension. (2013) 62:706–11. doi: 10.1161/HYPERTENSIONAHA.113.01414 23940194 PMC4085393

[18] Harbaoui B Courand PY Milon H Fauvel JP Khettab F Mechtouff L . Association of various blood pressure variables and vascular phenotypes with coronary, stroke and renal deaths: Potential implications for prevention. Atherosclerosis. (2015) 243:161–8. doi: 10.1016/j.atherosclerosis.2015.09.011 26386213

[19] Thiagarajah R Kandasamy R Sellamuthu P. Hypertensive retinopathy and the risk of hemorrhagic stroke. J Korean Neurosurg Soc. (2021) 64:543–51. doi: 10.3340/jkns.2020.0285 34237912 PMC8273771

[20] Chen X Liu L Liu M Huang X Meng Y She H . Hypertensive retinopathy and the risk of stroke among hypertensive adults in China. Invest Ophthalmol Vis Sci. (2021) 62:28. doi: 10.1167/iovs.62.9.28 34283210 PMC8300046

[21] Jianyuan Z. Multivariate analysis of the risk of stroke in patients with hypertensive retinopathy. Cardiovasc Dis Electron J Integr Tradit Chin West Med. (2021) 9:40–2.

[22] Chengwu L Shangkun Z Jing L . Multivariate analysis of the risk of stroke in the patients with hypertensive retinopathy. Chin J Chin Ophthalmol. (2021) 31:337–40.

[23] Li J Kokubo Y Arafa A Sheerah HA Watanabe M Nakao YM . Mild hypertensive retinopathy and risk of cardiovascular disease: the suita study. 2*J Atheroscler Thromb*. (2022) 29:1663–71. doi: 10.5551/jat.63317 35034920 PMC9623077

[24] Petitti DB Bhatt H. Retinopathy as a risk factor for nonembolic stroke in diabetic subjects. Stroke. (1995) 26:593–6. doi: 10.1161/01.STR.26.4.593 7709403

[25] Klein BE Klein R McBride PE Cruickshanks KJ Palta M Knudtson MD . Cardiovascular disease, mortality, and retinal microvascular characteristics in type 1 diabetes: wisconsin epidemiologic study of diabetic retinopathy. Arch Intern Med. (2004) 164:1917–24. doi: 10.1001/archinte.164.17.1917 15451768

[26] Cheung N Rogers S Couper DJ Klein R Sharrett AR Wong TY. Is diabetic retinopathy an independent risk factor for ischemic stroke? Stroke. (2006) 38:398–401. doi: 10.1161/01.STR.0000254547.91276.50 17194880

[27] Hägg S Thorn LM Putaala J Liebkind R Harjutsalo V Forsblom CM . Incidence of stroke according to presence of diabetic nephropathy and severe diabetic retinopathy in patients with type 1 diabetes. Diabetes Care. (2013) 36:4140–6. doi: 10.2337/dc13-0669 24101700 PMC3836162

[28] Wong KH Hu K Peterson C Sheibani N Tsivgoulis G Majersik JJ . Diabetic retinopathy and risk of stroke: a secondary analysis of the ACCORD eye study. Stroke. (2020) 51:3733–6. doi: 10.1161/STROKEAHA.120.030350 33019896 PMC7686117

[29] Yang GR Li D Li L. Comparison of coronary heart disease and stroke in association with diabetic retinopathy in adults with diabetes using a national survey. Diabetes Metab Syndr Obes. (2020) 13:5079–84. doi: 10.2147/DMSO.S292934 33380817 PMC7767698

[30] Drinkwater JJ Davis TME Hellbusch V Turner AW Bruce DG Davis WA. Retinopathy predicts stroke but not myocardial infarction in type 2 diabetes: the Fremantle Diabetes Study Phase II. Cardiovasc Diabetol. (2020) 19:43. doi: 10.1186/s12933-020-01018-3 32234054 PMC7110810

[31] Hsu CY Lee CM Chou KY Lee CY Chen HC Chiou JY . The association of diabetic retinopathy and cardiovascular disease: a 13-year nationwide population-based cohort study. Int J Environ Res Public Health. (2021) 18:8106. doi: 10.3390/ijerph18158106 34360398 PMC8345672

[32] Modjtahedi BS Wu J Luong TQ Gandhi NK Fong DS Chen W. Severity of diabetic retinopathy and the risk of future cerebrovascular disease, cardiovascular disease, and all-cause mortality. Ophthalmology. (2021) 128:1169–79. doi: 10.1016/j.ophtha.2020.12.019 33359888

[33] Eriksson MI Hietala K Summanen P Harjutsalo V Putaala J Ylinen A . Stroke incidence increases with diabetic retinopathy severity and macular edema in type 1 diabetes. Cardiovasc Diabetol. (2024) 23:136. doi: 10.1186/s12933-024-02235-w 38664827 PMC11046873

[34] Tang J Huang P. The association in diabetic retinopathy and stroke finding from NHANES evidence. Int Ophthalmol. (2024) 44:170. doi: 10.1007/s10792-024-03098-6 38587685

[35] Grunwald JE Ying GS Maguire M Pistilli M Daniel E Alexander J . Association between retinopathy and cardiovascular disease in patients with chronic kidney disease (from the Chronic Renal Insufficiency Cohort [CRIC] Study). Am J Cardiol. (2012) 110:246–53. doi: 10.1016/j.amjcard.2012.03.014 22516527 PMC3383900

[36] Grunwald JE Pistilli M Ying GS Maguire M Daniel E Whittock-Martin R . Retinopathy and the risk of cardiovascular disease in patients with chronic kidney disease (from the Chronic Renal Insufficiency Cohort study). Am J Cardiol. (2015) 116:1527–33. doi: 10.1016/j.amjcard.2015.08.015 26409637 PMC4630087

[37] Wong TY Klein R Sharrett AR Couper DJ Klein BE Liao DP . Cerebral white matter lesions, retinopathy, and incident clinical stroke. JAMA. (2002) 288:67–74. doi: 10.1001/jama.288.1.67 12090864

[38] Tang Q Zhang Y Yang Z Li S Wu M Guo Y . Study on the interaction between the characteristics of retinal microangiopathy and risk factors for cerebral small vessel disease. Contrast Media Mol Imaging. (2022) 2022:9505945. doi: 10.1155/2022/9505945 35800241 PMC9203197

[39] Wang L Shah S Llaneras CN Goldhardt R. Insight into the brain: application of the retinal microvasculature as a biomarker for cerebrovascular diseases through optical coherence tomography angiography. Curr Ophthalmol Rep. (2024) 12:1–11. doi: 10.1007/s40135-023-00320-z 39310044 PMC11415260

[40] Feuer DS Handberg EM Mehrad B Wei J Bairey Merz CN Pepine CJ . Microvascular dysfunction as a systemic disease: a review of the evidence. Am J Med. (2022) 135:1059–68. doi: 10.1016/j.amjmed.2022.04.006 35472396 PMC9427712

[41] Shah W Gong Y Qiao X Lu Y Ding Y Zhang Z . Exploring endothelial cell dysfunction's impact on the brain-retina microenvironment connection: molecular mechanisms and implications. Mol Neurobiol. (2025) 62:7484–505. doi: 10.1007/s12035-025-04714-x 39904964

[42] Dumitrascu OM Demaerschalk BM Valencia Sanchez C Almader-Douglas D O'Carroll CB Aguilar MI . Retinal microvascular abnormalities as surrogate markers of cerebrovascular ischemic disease: a meta-analysis. J Stroke Cerebrovasc Dis. (2018) 27:1960–8. doi: 10.1016/j.jstrokecerebrovasdis.2018.02.041 29571764

[43] White T Selvarajah V Wolfhagen-Sand F . Prediction of cardiovascular risk factors from retinal fundus photographs: Validation of a deep learning algorithm in a prospective non-interventional study in Kenya. Diabetes Obes Metab. (2024) 26:2722–31. 38618987 10.1111/dom.15587

[44] Yusufu M Friedman DS Kang M Padhye A Shang X Zhang L . Retinal vascular fingerprints predict incident stroke: findings from the UK Biobank cohort study. Heart. (2025) 111:306–13. doi: 10.1136/heartjnl-2024-324705 39805634

