# Supplementary material for: Meta-analysis of the association between retinopathy and the risk of stroke
Source: Front Med (Lausanne). 2026 Jun 30;13:1887366. doi: 10.3389/fmed.2026.1887366 (PMC13365340; doi:10.3389/fmed.2026.1887366)
Supplement: Supplementary file 1 [file Supplementary_file_1.docx]

Table S1

| **Database** | **Detailed Search Strategy** |
| --- | --- |
| PubMed | #1 "Retinopathy"[Mesh]  #2 "Diabetic Retinopathy"[Mesh]  #3 retinopathy[Title/Abstract]  #4 diabetic retinopathy[Title/Abstract]  #5 hypertensive retinopathy[Title/Abstract]  #6 retinal microvascular abnormalit*[Title/Abstract]  #7 retinal vascular abnormalit*[Title/Abstract]  #8 retinal microvascul*[Title/Abstract]  #9 OR/#1-#8  #10 "Stroke"[Mesh]  #11 "Cerebrovascular Disorders"[Mesh]  #12 stroke[Title/Abstract]  #13 cerebrovascular disease*[Title/Abstract]  #14 cerebrovascular disorder*[Title/Abstract]  #15 cerebral infarction[Title/Abstract]  #16 ischemic stroke[Title/Abstract]  #17 ischaemic stroke[Title/Abstract]  #18 hemorrhagic stroke[Title/Abstract]  #19 haemorrhagic stroke[Title/Abstract]  #20 brain infarction[Title/Abstract]  #21 OR/#10-#20  #22 #9 AND #21 |
| Embase | ('retinopathy'/exp OR 'diabetic retinopathy'/exp OR retinopathy:ti,ab OR 'diabetic retinopathy':ti,ab OR 'hypertensive retinopathy':ti,ab OR 'retinal microvascular abnormality':ti,ab OR 'retinal vascular abnormality':ti,ab) AND ('stroke'/exp OR 'cerebrovascular disease'/exp OR stroke:ti,ab OR 'cerebrovascular disease':ti,ab OR 'cerebral infarction':ti,ab OR 'ischemic stroke':ti,ab OR 'haemorrhagic stroke':ti,ab) AND [humans]/lim |
| Cochrane Library | (retinopathy OR diabetic retinopathy OR hypertensive retinopathy OR retinal microvascular abnormalities) AND (stroke OR cerebrovascular disease OR cerebral infarction OR ischemic stroke OR haemorrhagic stroke) |
| CNKI | 主题 =("视网膜病变" OR "糖尿病视网膜病变" OR "高血压视网膜病变") AND ("脑卒中" OR "卒中" OR "脑血管病" OR "脑梗死" OR "脑出血") |

Table S2

| **Study** | **Participants** | **Grading criteria for retinopathy** | **Main comparison** | **Effect estimate** | **Direction of association** |
| --- | --- | --- | --- | --- | --- |
| Ong | Hypertension | Wong and Mitchell classification | Mild vs None  Moderate-severe vs None | HR=1.35(0.96–1.89)  HR=2.37(1.39–4.02) | Positive/NS |
| Thiagarajah | Hypertension | Wong and Mitchell classification | Moderate-severe vs Mild-none | No regression analysis | — |
| Harbaoui | Hypertension | KWB classification | Grade1-2 vs None  Grade3-4 vs None | HR=1.99(0.99-4.00)  HR=3.35(1.52-7.38) | Positive/NS |
| Chen | Hypertension | KWB classification | Grade1-2 vs None  Grade3-4 vs None | HR=1.26(1.01-1.58)  HR=2.40(1.49-3.84) | Positive |
| Zhao | Hypertension | KWB classification | Grade4 vs Grade2 | OR=2.58(1.78-6.54) | Positive |
| Li | Hypertension | KWB classification | Grade3 vs Grade2  Grade4 vs Grade2 | OR=1.463(Not reported)  OR=1.682(Not reported) | — |
| Li | Hypertension | KWB classification | Grade1-2 vs None | HR=1.28(1.01–1.62) | Positive |
| Modjtahedi | Diabetes | ETDRS scale | Mild NPDR vs None  Moderate–severe NPDR vs None  PDR vs None | RR=1.44 (1.30-1.59)  RR=1.75(1.45-2.11)  RR=2.67 (1.95-3.66) | Positive |
| Eriksson | Diabetes | ETDRS scale | NPDR vs Mild-none  PDR vs Mild-none | HR=1.79 (1.02–3.15)  HR=1.69 (1.02–2.82) | Positive |
| Tang | Diabetes | ETDRS scale | NPDR vs None  PDR vs None | OR=1.61 (1.04–2.44)  OR=6.27(1.78–19.66) | Positive |
| Grunwald | Renal insufficiency | ETDRS scale | Mild NPDR vs None  Moderate–severe NPDR vs None  PDR vs None  Ungraded vs None | OR=1.58(0.92–2.71)  OR=1.17 (0.69–1.98)  OR=2.43 (1.43–4.15)  OR=1.65 (0.93–2.93) | Positive/NS |
| Grunwald | Renal insufficiency | ETDRS scale | Mild NPDR vs None  Moderate–severe NPDR vs None  PDR vs None  Ungraded vs None | Not estimable due to insufficient events  HR=3.17 (0.70–14.4)  HR=9.09 (2.18–37.8)  HR=6.49(1.14–37.0) | Positive/NS |
| Hughes | General adults | NHSDESP classification | Background retinopathy vs None  Pre-proliferative retinopathy vs None | OR=1.41 (1.01–1.96)  OR=2.79 (1.30–5.99) | Positive |

Table S3

| **Study** | **Total sample** **(n)** | **Stroke events** **(n)** | **Event rate (%)** | **Effect measure** |
| --- | --- | --- | --- | --- |
| Wong2022 | 2828 | 117 | 4.1 | HR |
| Drinkwater2020 | 1473 | 53 | 3.6 | HR |
| Cheung2006 | 1546 | 75 | 4.9 | HR |
| Klein2004 | 918 | 55 | 6.0 | OR |
| Hsu2021 | 13220 | NR | NR | HR |
| Wong2002 | 1684 | 32 | 1.9 | RR |
| Yang2020 | 21049 | 2055 | 9.8 | OR |
| Hägg2013 | 4083 | 149 | 3.6 | HR |
| Petitti1995 | 104 | NA | NA | RR |
| Suri2008 | 4753 | 309 | 6.5 | RR |

NR = not reported in the original study; NA = not applicable because the study used a case-control design and incidence rates could not be estimated.

Table S4

| **Section and Topic** | **Item #** | **Checklist item** | **Location where item is reported** |
| --- | --- | --- | --- |
| **TITLE** | | |  |
| Title | 1 | Identify the report as a systematic review. | Title page: Meta-analysis of the Association Between Retinopathy and the Risk of Stroke |
| **ABSTRACT** | | |  |
| Abstract | 2 | See the PRISMA 2020 for Abstracts checklist. | Abstract |
| **INTRODUCTION** | | |  |
| Rationale | 3 | Describe the rationale for the review in the context of existing knowledge. | Introduction, paragraphs 1–3 |
| Objectives | 4 | Provide an explicit statement of the objective(s) or question(s) the review addresses. | End of Introduction |
| **METHODS** | | |  |
| Eligibility criteria | 5 | Specify the inclusion and exclusion criteria for the review and how studies were grouped for the syntheses. | Methods: Inclusion and exclusion criteria |
| Information sources | 6 | Specify all databases, registers, websites, organisations, reference lists and other sources searched or consulted to identify studies. Specify the date when each source was last searched or consulted. | Methods: Search Strategy |
| Search strategy | 7 | Present the full search strategies for all databases, registers and websites, including any filters and limits used. | Supplementary Materials: Table S1 |
| Selection process | 8 | Specify the methods used to decide whether a study met the inclusion criteria of the review, including how many reviewers screened each record and each report retrieved, whether they worked independently, and if applicable, details of automation tools used in the process. | Methods: Inclusion and exclusion criteria  Methods: Data Extraction and Quality Assessment |
| Data collection process | 9 | Specify the methods used to collect data from reports, including how many reviewers collected data from each report, whether they worked independently, any processes for obtaining or confirming data from study investigators, and if applicable, details of automation tools used in the process. | Methods: Data Extraction and Quality Assessment |
| Data items | 10a | List and define all outcomes for which data were sought. Specify whether all results that were compatible with each outcome domain in each study were sought (e.g. for all measures, time points, analyses), and if not, the methods used to decide which results to collect. | Methods: Data Extraction and Quality Assessment |
|  | 10b | List and define all other variables for which data were sought (e.g. participant and intervention characteristics, funding sources). Describe any assumptions made about any missing or unclear information. | Methods: Data Extraction and Quality Assessment |
| Study risk of bias assessment | 11 | Specify the methods used to assess risk of bias in the included studies, including details of the tool(s) used, how many reviewers assessed each study and whether they worked independently, and if applicable, details of automation tools used in the process. | Methods: Data Extraction and Quality Assessment |
| Effect measures | 12 | Specify for each outcome the effect measure(s) (e.g. risk ratio, mean difference) used in the synthesis or presentation of results. | Methods: Statistical Analysis |
| Synthesis methods | 13a | Describe the processes used to decide which studies were eligible for each synthesis (e.g. tabulating the study intervention characteristics and comparing against the planned groups for each synthesis (item #5)). | Methods: Statistical Analysis |
|  | 13b | Describe any methods required to prepare the data for presentation or synthesis, such as handling of missing summary statistics, or data conversions. | Methods: Statistical Analysis |
|  | 13c | Describe any methods used to tabulate or visually display results of individual studies and syntheses. | Methods: Statistical Analysis |
|  | 13d | Describe any methods used to synthesize results and provide a rationale for the choice(s). If meta-analysis was performed, describe the model(s), method(s) to identify the presence and extent of statistical heterogeneity, and software package(s) used. | Methods: Statistical Analysis |
|  | 13e | Describe any methods used to explore possible causes of heterogeneity among study results (e.g. subgroup analysis, meta-regression). | Methods: Statistical Analysis |
|  | 13f | Describe any sensitivity analyses conducted to assess robustness of the synthesized results. | Methods: Statistical Analysis |
| Reporting bias assessment | 14 | Describe any methods used to assess risk of bias due to missing results in a synthesis (arising from reporting biases). | Methods: Statistical Analysis |
| Certainty assessment | 15 | Describe any methods used to assess certainty (or confidence) in the body of evidence for an outcome. | Not performed |
| **RESULTS** | | |  |
| Study selection | 16a | Describe the results of the search and selection process, from the number of records identified in the search to the number of studies included in the review, ideally using a flow diagram. | Results: Study Selection and Study Characteristics  Results: Figure 1 |
|  | 16b | Cite studies that might appear to meet the inclusion criteria, but which were excluded, and explain why they were excluded. | Results: Qualitative synthesis of studies not included in the meta-analysis  Supplementary Materials: Table S2 |
| Study characteristics | 17 | Cite each included study and present its characteristics. | Results: Tables 1–2 |
| Risk of bias in studies | 18 | Present assessments of risk of bias for each included study. | Results: Tables 1–2 |
| Results of individual studies | 19 | For all outcomes, present, for each study: (a) summary statistics for each group (where appropriate) and (b) an effect estimate and its precision (e.g. confidence/credible interval), ideally using structured tables or plots. | Results: Tables 1–2  Results: Figure 1  Supplementary Materials: : Table S2 |
| Results of syntheses | 20a | For each synthesis, briefly summarise the characteristics and risk of bias among contributing studies. | Results: Tables 1 |
|  | 20b | Present results of all statistical syntheses conducted. If meta-analysis was done, present for each the summary estimate and its precision (e.g. confidence/credible interval) and measures of statistical heterogeneity. If comparing groups, describe the direction of the effect. | Results: Synthesis of Results; Figure 2; Table 3 |
|  | 20c | Present results of all investigations of possible causes of heterogeneity among study results. | Results: Synthesis of Results; Table 3 |
|  | 20d | Present results of all sensitivity analyses conducted to assess the robustness of the synthesized results. | Results: Sensitivity Analysis; Figure 3 |
| Reporting biases | 21 | Present assessments of risk of bias due to missing results (arising from reporting biases) for each synthesis assessed. | Results: Publication Bias; Figure 4 |
| Certainty of evidence | 22 | Present assessments of certainty (or confidence) in the body of evidence for each outcome assessed. | Not performed |
| **DISCUSSION** | | |  |
| Discussion | 23a | Provide a general interpretation of the results in the context of other evidence. | Discussion, paragraphs 1–4 |
|  | 23b | Discuss any limitations of the evidence included in the review. | Discussion: Limitations paragraph |
|  | 23c | Discuss any limitations of the review processes used. | Discussion: Limitations paragraph |
|  | 23d | Discuss implications of the results for practice, policy, and future research. | Discussion; Conclusion |
| **OTHER INFORMATION** | | |  |
| Registration and protocol | 24a | Provide registration information for the review, including register name and registration number, or state that the review was not registered. | Methods: Statistical Analysis  Discussion: Limitations |
|  | 24b | Indicate where the review protocol can be accessed, or state that a protocol was not prepared. | Methods: Statistical Analysis |
|  | 24c | Describe and explain any amendments to information provided at registration or in the protocol. | Not applicable |
| Support | 25 | Describe sources of financial or non-financial support for the review, and the role of the funders or sponsors in the review. | Funding Statement |
| Competing interests | 26 | Declare any competing interests of review authors. | Conflict of Interest |
| Availability of data, code and other materials | 27 | Report which of the following are publicly available and where they can be found: template data collection forms; data extracted from included studies; data used for all analyses; analytic code; any other materials used in the review. | Data availability statement |

Figure S1
